# Mono- and Multi-Objective CFD Optimization of Graded Foam-Filled Channels

**DOI:** 10.3390/ma15030968

**Published:** 2022-01-27

**Authors:** Gerardo Maria Mauro, Marcello Iasiello, Nicola Bianco, Wilson K. S. Chiu, Vincenzo Naso

**Affiliations:** 1 Dipartimento di Ingegneria, Università degli Studi del Sannio, Piazza Roma 21, 82100 Benevento, Italy; germauro@unisannio.it; 2 Dipartimento di Ingegneria Industriale, Università degli Studi di Napoli Federico II, Piazzale Tecchio 80, 80125 Napoli, Italy; nicola.bianco@unina.it (N.B.); vincenzo.naso@unina.it (V.N.); 3 Department of Mechanical Engineering, University of Connecticut, 191 Auditorium Road, Storrs, CT 06269, USA; wilson.chiu@uconn.edu

**Keywords:** heat sinks, porous media, thermal management, graded foams, genetic algorithms, mono- and multi-objective optimization

## Abstract

Graded foam-filled channels are a very promising solution for improving the thermal performance of heat sinks because of their customized structures that leave large amounts of room for heat transfer enhancement. Accordingly, this paper proposes a comprehensive optimization framework to address the design of such components, which are subjected to a uniform heat flux boundary condition. The graded foam is achieved by parameterizing the spatial distributions of porosity and/or Pores Per Inch (PPI). Mono- and multi-objective optimizations are implemented to find the best combination of the foam’s fluid-dynamic, geometrical and morphological design variables. The mono-objective approach addresses the Performance Evaluation Criterion (PEC) as an objective function to maximize the thermal efficiency of graded foams. The multi-objective approach addresses different objective functions by means of Pareto optimization to identify the optimal tradeoff solutions between heat transfer enhancement and pressure drop reduction. Optimizations are performed by assuming a local thermal non-equilibrium in the foam. They allowed us to achieve a 1.51 PEC value with H* = 0.50, Re_H_ = 15000, *i_ε_* = i_PPI_ = 0.50, *ε*(0) = 0.85, *ε*(1) = 0.97, PPI(0) = 5, PPI(1) = 40, and *k_s__→f_* = 10^4^ as the design variables. For the three multi-objective functions investigated, one can extrapolate the optimum from the Pareto front via the utopia criterion, obtaining $\bar{h}$ = 502 W/m^2^ K and Δ*p* = 80 Pa, $\bar{{\mathrm{Nu}}_{H,\mathrm{unif}}}$ = 2790 and *f* = 42, $\bar{{\langle T_{s}^{*}\rangle }^{s}}$= 0.011, and Δp* = 91. The optimal solutions provide original insights and guidelines for the thermal design of graded foam-filled channels.

## 1. Introduction

Open-cell foams are a very promising kind of material for enhancing heat transfer. They are porous materials that are also known as cellular materials because they consist of many communicating cells that are periodically repeated through the space. Because of their relatively high effective thermal conductivity and heat transfer area to volume ratio, as well as of their tortuosity characteristics that promote flow mixing, they are highly effective for applications where heat transfer plays a primary role, such as heat sinks [1,2], thermal energy storage systems [3], nanofluids-based heat exchangers [4], and so on.

The heat transfer characteristics of foams are strongly dependent on their microstructure. Therefore, in recent years, many solutions, such as customized foams obtained with additive manufacturing [5,6], sintered [7] or stacked [8] foam layers, or similar, have been proposed. All of them can be labeled as functionally graded foams [9,10,11], where foam characteristics, such as porosity or Pores Per Inch (PPI), vary through the foam sample.

Graded foams are promising in heat transfer applications, such as phase change materials [12], volumetric solar air receivers [13,14], and channels which are components of heat exchangers. Variable pore size foams were found to increase heat transfer coefficients if one assumes larger pore sizes in the first cells of the channel [15]. Pipes either partially or fully filled with graded foams under different PPIs and porosities, investigated by Wang et al. [16], showed enhanced heat transfer properties for decreasing cell sizes along the radial direction in channels that were partially filled. Xu and Gong [17] analyzed channels partially filled with graded foams and showed a decrease in the Nusselt number for both porosity and PPI increase, while the friction factor was more affected by PPI than by porosity variation. An analytical investigation of graded foams with a uniform heat flux orthogonal to the flow direction was carried out by Bai et al. [18]. The authors assumed the porosity to vary in the heat flux direction according to a quadratic law and showed that the best thermal performance was attained in foams with smaller porosities close to the heat source. Chen et al. [19] showed numerically that in a double-pipe heat exchanger, the best solution was to employ smaller porosities and PPIs at both sides of the inner pipe. Iasiello et al. [20] performed a parametric analysis of different porosities and/or PPI distributions in a channel heated from below and equipped with a foam. The Performance Evaluation Criteria (PEC) of graded foams were compared to those of uniform foams with porosities and PPIs the same as their averaged values; increases of up to 38% in the PEC with variable porosity and up to 42% with both variable porosity and PPI were found. This means that a graded foam would outperform a uniform-properties foam of these percentages if, for instance, friction power is constrained. Nonetheless, though these values are promising, they have been obtained through standard parametric analysis without finding optimal values because of the very high computational effort required.

Because of the increased computational power, Computational Fluid Dynamics (CFD) techniques, coupled with numerical optimization algorithms, such as genetic algorithms, have been widely used recently to perform mono- or multi-objective optimization analyses. In mono-objective analyses, optimum solutions are found by employing just one objective function; on the other hand, in multi-objective analyses, two or more contrasting objective functions are employed in a multiple-criteria decision analysis to obtain a set of optimal solutions. Objective functions based on heat transfer and pressure drop, which are always in contrast, have often been employed. Safikhani et al. [21] employed a genetic algorithm to address heat transfer and pressure drop in helically corrugated tubes by using both geometrical and fluid-dynamic design variables. Chamoli et al. [22] derived a scaled Nusselt number as a function of the friction factor Pareto front for a heat exchanger tube fitted with compound insert geometries. Liu et al. [23] employed the Colburn j-factor and the friction factor as the objective functions in a plate-fin heat exchanger for the hydraulic retarder. The literature survey highlights that the above said objective functions are commonly employed in the heat exchanger optimizations and that CFD techniques are used to figure out the investigated problem. Optimization was carried out by employing geometrical characteristics as design variables. The results showed a 12.8% increase in the Colburn j-factor and a 26.9% decrease in the friction factor. Optimization techniques in porous materials for volumetric solar receivers have also been employed [24,25]. A mono-objective optimization to maximize the PEC in a heated tube was performed by Zheng et al. [26] by employing multiple-layer porous material inserts with different porosities. A 2.5 factor increase in the PEC, compared to a single foam layer, was found in a graded foam. Siavashi et al. [27] optimized devices equipped with graded foams and nanoparticles by employing various porosities and particle sizes as the design variables. A particle swarm optimization algorithm with the PEC as the single-objective function achieved an up to 2.5% increase in the PEC. Bianco et al. [28] optimized the heat rate and the pumping power in finned and unfinned heat sinks equipped with metal foams, obtaining a five to six factor increase in the heat rate compared to available experimental data [29], at constrained pumping power. Shi et al. [30] optimized a graded porous medium partially filling a tube, assuming linearly variable pore sizes and porosities and employing the Nusselt number and the friction factor as the objective functions. The authors found that decreasing the pore size affected heat transfer more than lowering the porosity, whereas the effects of increasing porosities at a larger channel filling ratio were more marked. The optimization provided a 19.6% reduction in the pressure drop and an up to 7.1% increase compared to that in homogeneous porous media.

The literature survey shows that, because of their customized structure, graded porous materials are very promising for heat transfer enhancement. Moreover, the highly useful prediction of the best mix of design variables, such as porosity and PPI, as well as of their distribution through the investigated domain, can be performed only with numerical optimization methods because of the large computational effort required. This implies that, nowadays, optimizing the performance of heat transfer devices requires the combination of customized porous materials and numerical tools, as well as realizing how these newly derived solutions are helpful to thermal management.

In this work, CFD mono- and multi-objective optimizations are performed, in order to evaluate the fluid-dynamic, geometrical and morphological design variables of a graded foam that maximize heat transfer and minimize pressure drop. The mathematical model is set up with reference to a local thermal non-equilibrium in the foam. A uniform laminar steady-state condition is assumed based on transient and turbulence effect investigations [31,32], while a uniform heat flux boundary condition is assumed at one side of the domain. Optimization is carried out with a non-dominated sorting genetic algorithm (NSGA-II) for both mono-objective optimization, where the PEC is used as objective function, and for multi-objective optimization, where Pareto fronts are obtained to address different objective functions related to the heat transfer—pressure drop dualism. Accordingly, the main originalities of this work are:

- To propose a comprehensive optimization approach to the design of graded foams with a continuous distribution of the design variables (porosity and PPI) in order to enhance the thermal performance of heat sinks; such a framework would enable the exploration of new additive manufacturing technologies that are capable of building up arbitrary geometries with acceptable cost;
- To compare different multi-objective approaches in order to find the best tradeoff solutions between heat transfer enhancement and pressure drop reduction.

## 2. Mathematical Modeling

### 2.1. Governing Equations

**Figure 1:**
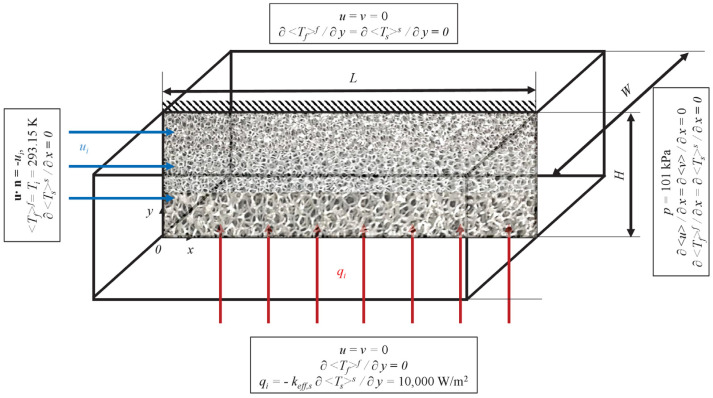
The domain of a graded foam, with PPI increasing along the height, and the problem boundary conditions.

The graded metal foam with variable porosity and/or variable PPI investigated in the present paper is sketched in Figure 1. Its thickness, *H*, and length, *L*, are assumed to be negligible compared to its width, *W*. Heat transfer and pressure drop in a casted metal foam with spatial distributions of porosity and/or PPI will be optimized in the following.

As in Iasiello et al. [20], we assume porosity, *ε*, and PPI varying according to a power law with indices *i_ε_* and *i*_PPI_*,* with values at the boundaries (y* = *H*/*L* = 0 and y* = 1) typical in commercial metal foams, say *ε* = 0.85, 0.97 and PPI = 5, 40:

(1) $$\varepsilon \left(y^{*}\right)=\left[\varepsilon \left(1\right)-\varepsilon \left(0\right)\right]y^{{*}^{i_{\varepsilon }}}+\varepsilon \left(0\right),$$

(2) $$PPI\left(y^{*}\right)=\left[PPI\left(1\right)-PPI\left(0\right)\right]y^{{*}^{i_{PPI}}}+PPI\left(0\right),$$

On the other hand, graded foams are also compared to uniform foams with porosity and PPI the same as their averaged values, $\bar{\varepsilon }$ and $\bar{PPI}$, computed via the following correlations:

(3) $$\bar{\varepsilon }=\frac{\varepsilon \left(1\right)-\varepsilon \left(0\right)}{i_{\varepsilon }+1}+\varepsilon \left(0\right),$$

(4) $$\bar{PPI}=\frac{PPI\left(1\right)-PPI\left(0\right)}{i_{PPI}+1}+PPI\left(0\right),$$

**Figure 2:**
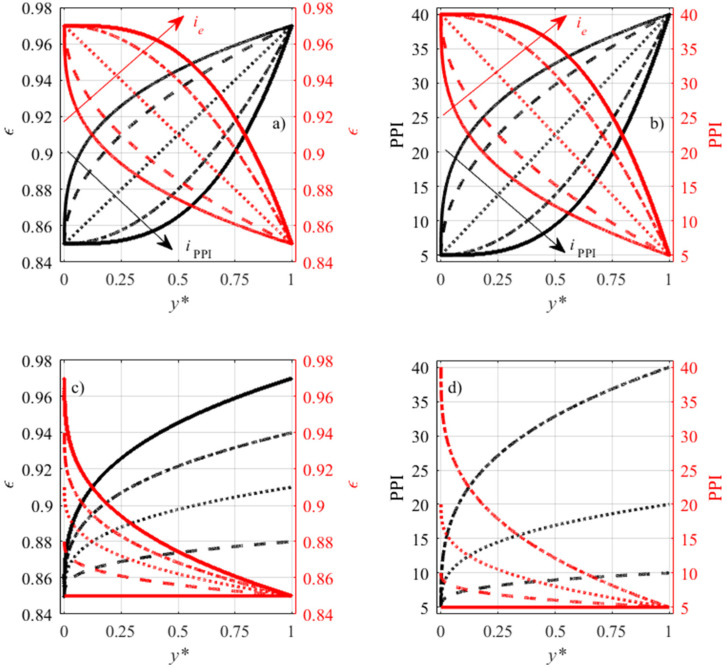
Variable porosity and PPI functions obtained via Equations (1) and (2): (**a**,**b**) *i* = 0.33, 0.50, 1.00, 2.00, 3.00; (**c**,**d**): *i* = 0.33.

The characteristics functions evaluated with Equations (1) and (2), for different values of the index *i*, and for different *ε*(1), *ε*(0), PPI(1), and PPI(0), are presented in Figure 2.

Because of the complex geometry, the volume averaging technique [33] is herein employed. According to this technique, governing equations are written over a Representative Elementary Volume (REV) of the foam [34], where each averaged variable is signed in brackets < > and assumed to be its volume average over the REV. Additionally, if, during this volume averaging process, the local temperature difference is relevant, then one could employ a local thermal non-equilibrium model [35], where the energy equation for each phase is written with a source term that accounts for the interfacial convective heat transfer between the two phases.

The volume-averaged equations for a porous medium with variable characteristics, under the assumptions of laminar incompressible steady-state flow, negligible buoyancy, radiation, thermal dispersion, uniform thermophysical properties, and local thermal non-equilibrium between the two phases, are the same as those in [14,35,36,37]:

(5) ∇⋅〈u〉=0,

(6) $$\frac{\rho_{f}}{\varepsilon }\left(\langle u\rangle \nabla \cdot \langle u\rangle \right)=-\nabla {\langle p\rangle }^{f}+\frac{\mu_{f}}{\varepsilon }\nabla^{2}\langle u\rangle -\frac{\mu_{f}}{K}\langle u\rangle -\frac{\rho_{f}\;C_{f}\;\varepsilon }{\sqrt{K}}\left|\langle u\rangle \right|\langle u\rangle ,$$

(7) $${\left(\rho \;c_{p}\right)}_{f}\langle u\rangle \cdot \nabla {\langle T_{f}\rangle }^{f}=\nabla \cdot \left(k_{eff,f}\;\nabla {\langle T_{f}\rangle }^{f}\right)+h_{v}\left({\langle T_{s}\rangle }^{s}-{\langle T_{f}\rangle }^{f}\right)$$

(8) $$\nabla \cdot \left(k_{eff,s}\;\nabla {\langle T_{s}\rangle }^{s}\right)-h_{v}\left({\langle T_{s}\rangle }^{s}-{\langle T_{f}\rangle }^{f}\right)=0,$$

with **u** is the velocity vector, *ρ* is the density, *p* is the pressure, *μ* is the dynamic viscosity, *K* is the permeability, *C_f_* is the inertial factor, *c_p_* is the specific heat capacity, *T* is the temperature, *k_eff_* is the effective thermal conductivity, and *h_v_* is the volumetric convection heat transfer coefficient.

The effects of either natural or mixed convection can be neglected, since the 0.28 maximum value of the Richardson number, based on a uniform heat flux Grashof number [38], with the cell size as the characteristic length [39,40] and the pore velocity as the velocity-restrictive case herein investigated, is far smaller than 10, the typical forced-mixed convection transition value for a porous media [41]. On the other hand, turbulence effects are negligible, too. In fact, though in the worst case the cell size-based Reynolds number achieved is roughly 670, that is higher than typical transition values [42], and so turbulence effects on both pressure drop [43] and convective heat transfer [44,45] can be neglected.

### 2.2. Closure Coefficients, Boundary Conditions and Numerical Modeling

In order to close Equations (5)–(8) and to guarantee the uniqueness of the solutions, porous media closure coefficients and boundary conditions are required. It is assumed that the closure coefficients depend on both porosity and foam cell size; thus, they depend on the coordinate y*. They are shown in Table 1, with their mathematical expression and sources. The coefficients in the momentum equations are taken from Calmidi [46]; the correlation between the cell size and PPI was given by Andreozzi et al. [47]; an isotropic thermal conductivity of both fluid and solid phases is assumed [48]; the volumetric heat transfer coefficients, the Reynolds and Nusselt numbers, are derived by neglecting the thermal entrance effects in the equations proposed by Iasiello et al. [49].

Boundary conditions, the same as those in Iasiello et al. [20], are reported in Figure 1, and are described in the following. As it is common for porous media, plug flow, together with a uniform temperature and no heat exchange with the environment (adiabatic) boundary condition, is assumed at the inlet section of the domain (x* = *x/L* = 0). A no-slip condition is invoked at the side walls (y* = 0 and y* = 1); a uniform heat flux, *q_i_*, through the bottom side wall (y* = 0) enters the solid phase of the foam, since *k_eff,s_*/*k_eff,f_* >> 1 (see [20]). The upper side wall (y* = 1) is assumed to be adiabatic. Finally, an atmospheric pressure and outflow condition is employed at the exit section of the domain (x* = 1).

Equations are solved with a standard numerical approach by means of a finite element code because the mathematical model does not require any particular techniques that are like meshless-based techniques [50,51]. For each run, 15,000 rectangular elements, 150 in the *y* direction, according to an arithmetic sequence, with a consecutive elements ratio of 10, and 100 in the *x* direction, were employed. Grid convergence for velocity, pressure, and temperature was verified, as in [20]. Up to 240,000 different grid elements with an equal arithmetic sequence ratio were analyzed by constraining the arithmetic sequence ratio. Namely, a number of elements that allows us to underline the channeling effect, as in [20], was employed for the velocity profile. Average pressure and temperature for both phases were checked with a 0.5% tolerance compared to the highest number of elements simulated in the grid convergence analysis. The check on the Nusselt number and friction factor was carried out, as in [20]; it showed that choosing more than 15,000 elements would not significantly improve the solution. It is worth underlining that the grid convergence plays a primary role, since many configurations were simulated in order to achieve optimum points. The fully coupled linear solver PARDISO was employed with a 10^−4^ RMS deviation. The herein employed model had been validated in [20] by comparing predicted dimensionless temperatures as a function of the axial coordinate, in different cross-sections, to experimental results from Dukan and Chen [52]; the agreement was very good.

### 2.3. Optimization Procedure and Data Reduction

**Figure 3:**
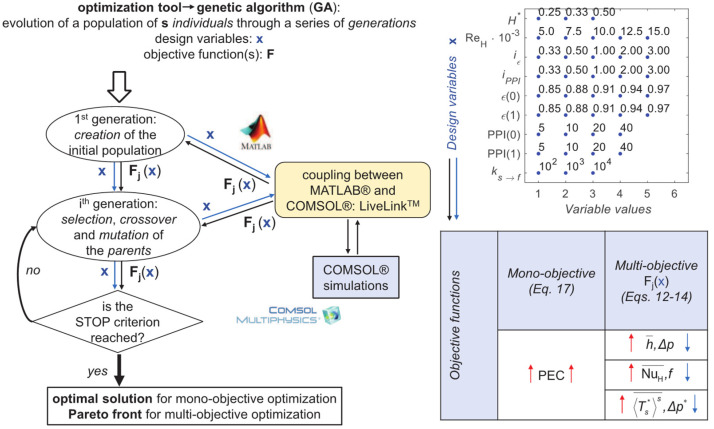
The optimization procedure, together with design variables **x** and objective functions **F_j_**(**x**).

The design of the graded foam-filled channel was optimized with different mono- and multi-objective approaches by implementing the procedure described in Figure 3, where a flowchart on its left side resumes the optimization procedure and the upward red and downward blue arrows refer to objective functions to be maximized and minimized, respectively. Mono- and multi-objective analyses are carried out in the following, with reference to a unit size of the domain in the *z* direction of the 2D problem under investigation. Because of the large solutions domain to be investigated, optimization is carried out by running a genetic algorithm (GA), coupling MATLAB^®^ R2018b and COMSOL Multiphysics^®^ (5.2). The model for the GA fitness function is developed in COMSOL Multiphysics, while COMSOL Multiphysics^®^ LiveLink^TM^ (5.2) for MATLAB^®^ is used for coupling the CFD solution with the GA in MATLAB^®^. A non-dominated sorting genetic algorithm (NSGA-II), which is a population-based numerical optimization technique, is implemented via the MATLAB^®^ functions *ga.m* for the mono- and *gamultiobj.m* for the multi-objective cases. Further details of the optimization algorithm can be found in [28]. The GA parameters for mono- and multi-objective optimizations are set according to MATLAB^®^ recommended values and authors’ expertise, as detailed below:

- The population size (number of solutions investigated by each iteration/generation) is assumed to be equal to 50 in both mono- and multi-objective optimizations;
- The crossover fraction is assumed to be equal to 0.60 in the mono-objective optimizations, and equal to 0.45 in the multi-objective ones;
- The mutation probability is assumed to be equal to 0.20 in both mono- and multi-objective optimizations;
- The maximum number of generations is assumed to be equal to 50 in both mono- and multi-objective optimizations, with a 0.1 tolerance criterion, i.e., the stop criterion depicted in Figure 3.

Notably, with the multi-objective optimizations, a two-objective approach addressing different couples of objective functions is implemented. A Pareto front collecting optimal non-dominated solutions is achieved for each couple. A comprehensive analysis of the solutions allows us to identify recurrent optimal values and, then, by applying the utopia point criterion [28] for multi-criteria decision making, a Pareto solution is chosen. The above said solution, denoted as a utopia optimum, represents the best trade-off among the objective functions. The utopia optimum is obtained by means of the following graphical construction. Once the Pareto front for a generic objective function with two performance indicators, *f*_1_ and *f*_2_, to be maximized and minimized, respectively, is obtained, one can define the utopia point, that is, a couple of values made up by the maximum and minimum values, *f*_1*,min*_ and *f*_2*,max*_, from the Pareto front. The utopia optimum is the couple of solutions (*f*_1*,opt*_, *f*_2*,opt*_) closer to the aforementioned utopia point (*f*_1,*min*_, *f*_2*,max*_).

Design variables and objective functions are presented in the right side of Figure 3. Porosity *ε* and PPI foam characteristics, at y* = 0 and y* = 1, variable through the foam domain via the indices *i_ε_* and *i*_PPI_ defined in Equations (1) and (2), are considered to be the discrete design variables, together with the following dimensionless variables:

(9) $$H^{*}=\frac{H}{L}$$

(10) $$Re_{H}=\frac{u_{i}\;H}{\nu }$$

(11) $$k_{s\to f}=\frac{k_{s}}{k_{f}}$$

The dimensionless foam height, H*, in Equation (9), was chosen to account for the geometrical characteristics of the channel. The Reynolds number, Re_H_, in Equation (10), refers to the macro-scale of the problem, the channel height, whereas the cell size is the pore micro-scale. Finally, the thermal conductivity ratio in Equation (11), *k_s→f_*, points out the preferential pattern of the heat through the porous medium.

One can deduce from Figure 3 that the solution domain comprises 450,000 variable combinations, i.e., solutions; each of them requires about 1 min computational time to be simulated via COMSOL Multiphysics^®^, using a 3.70 GHz 6-core processor Intel^®^ Core^™^ i7-8700K equipped with 32 GB Random-Access Memory (RAM). Finally, an exhaustive or brute-force search would require more than 300 days, which makes it unfeasible; it is also worth considering that the selected ranges of the design variables could be extended during the optimization.

Three couples of objective functions, including contrasting performance indicators, **F**_1_(**x**), **F**_2_(**x**), and **F**_3_(**x**), with **x** as the design variables vector, are considered in the multi-objective analysis:

(12) $$F_{1}\left(x\right)=\left\{ \begin{array}{l} \bar{h}=\frac{q_{e}}{\bar{{\langle T_{s}\rangle }^{s}}-\bar{\;{\langle T_{f}\rangle }^{f}}}\\ \Delta p=\frac{1}{H}\iint p\left(0,y\right)dy \end{array}\right.$$

(13) $$F_{2}\left(x\right)=\left\{ \begin{array}{l} \bar{Nu_{H}}=\frac{\bar{h}\;H}{\bar{k_{eff,f}}}=\frac{\bar{h}\;H}{\bar{\varepsilon }\;k_{f}}\\ f=\frac{\Delta p}{L}\frac{H}{\rho_{f}u_{i}^{2}/2} \end{array}\right.,$$

(14) $$F_{3}\left(x\right)=\left\{ \begin{array}{l} \bar{{\langle T_{s}^{*}\rangle }^{s}}=\frac{\bar{{\langle T_{s}\rangle }^{s}}-T_{i}}{q_{e}H/k_{eff,s}\left(\bar{\varepsilon }\right)}\\ \Delta p^{*}=\frac{\Delta p}{\frac{1}{2}\rho u_{i}^{2}} \end{array}\right.,$$

where:

(15) $$\bar{{\langle T_{s}\rangle }^{s}}=\frac{1}{L}\int_{0}^{L}{\langle T_{s}\rangle }^{s}\left(y=0\right)dx,$$

(16) $$\bar{{\langle T_{f}\rangle }^{f}}=\frac{1}{H\;L}\left[\int_{0}^{L}\left(\int_{0}^{H}{\langle T_{f}\rangle }^{f}dy\right)dx\right],$$

The effective thermal conductivities in Equations (13) and (14) are computed with the expressions reported in Table 1, making reference to the average porosity, $\bar{\varepsilon }$, evaluated with Equation (3), accounting for *ε*(0), *ε*(1), and *i_ε_* in each case.

The reason why the above reported couples of objective functions are chosen is that **F**_1_(**x**) allows us to grasp the physical meaning of the problem, since both heat transfer rate and pressure drop are somehow related to the overall thermal resistance and drag resistance. The couple **F**_2_(**x**) is the dimensionless form of the quantities in **F**_1_(**x**). Finally, the dimensionless temperature and pressure drop in **F**_3_(**x**) make them meaningful in many applications, such as in electronics, where the main objective is to improve the thermal management of a surface by reducing its temperature when the heat to be removed is known.

Mono-objective analysis is performed by making reference to the Performance Evaluation Criterion (PEC), proposed by Webb and Eckert [53] and already used in [20]:

(17) $$\mathrm{PEC}=\frac{\left(\bar{{\mathrm{Nu}}_{H}}/\bar{{\mathrm{Nu}}_{H,\mathrm{unif}}}\right)}{{\left(f/f_{unif}\right)}^{1/3}},$$

where $\bar{{\mathrm{Nu}}_{H,\mathrm{unif}}}$ is the Nusselt number and *f_unif_* is the friction factor in a foam with uniform characteristics. They are computed by assuming uniform values of the porosity and PPI equal to their average values computed via Equations (3) and (4) for each computed case. According to Webb and Eckert [53], when PEC is higher than 1, the thermal performance of a foam with variable characteristics is better than that of foams with uniform characteristics and the same surface area at equal pumping power.

## 3. Results and Comments

### 3.1. Mono-Objective Optimization

**Figure 4:**
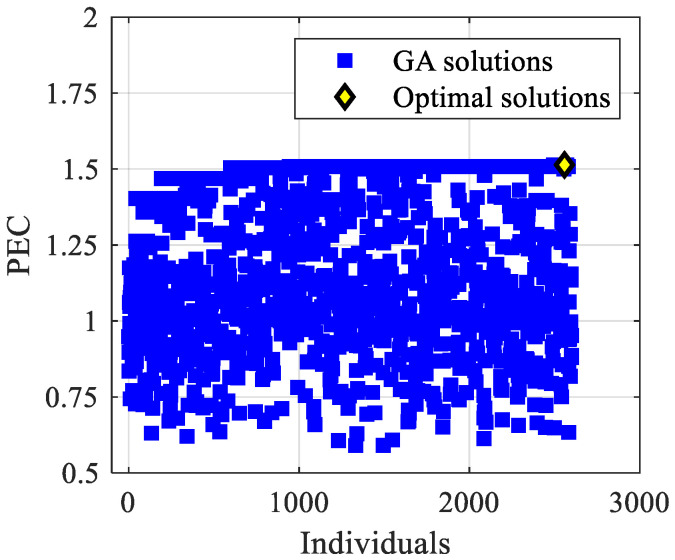
PEC vs. individuals for the mono-objective optimization case.

The mono-objective optimization aims to find the combination of the design variables that maximizes the PEC. The Performance Evaluation Criterion as a function of the individuals (i.e., *simulated solutions*), for mono-objective optimization, is reported in Figure 4. The figure shows that, above a certain number of simulations, the solutions investigated by the GA approach a 1.51 value. Data are scattered because of the large number of mutated individuals achieved for each generation. Notably, in order to avoid local minima, a quite high 0.20 mutation probability, amplified by the large solutions domain herein explored, was set. The 1.51 PEC was obtained with the following values of the design variables: H* = 0.33, *i_ε_* = 0.33, *i*_PPI_ = 1.00, Re_H_ = 15,000, *ε*(1) = 0.97, *ε*(0) = 0.85, PPI(1) = 40, PPI(0) = 5, and *k_s__→f_* = 1000. The foam morphology is rather consistent with that investigated by Iasiello et al. [20], who found a 1.42 maximum PEC with H* = 0.50, *i_ε_* = *i*_PPI_ = 0.50, Re_H_ = 15,000, *ε*(1) = 0.97, *ε*(1) = 0.85, PPI(1) = 40, PPI(0) = 5, and *k_s__→f_* = 10,000. The above said increase in the PEC was attained because the present optimization allowed us to investigate a larger number of cases than a standard sensitive analysis. Iasiello et al. [20] obtained the highest PEC by assuming equal indices for porosity and PPI. The herein simulation achieved a 9% increase in the PEC, compared to the 6% increase in [20], highlighting how optimization is better than a sensitivity analysis and showing that a numerical optimization, such as the one herein carried out with the genetic algorithm, is mandatory if exhaustive research requires much time and high cost.

### 3.2. Multi-Objective Optimization

**Figure 5:**
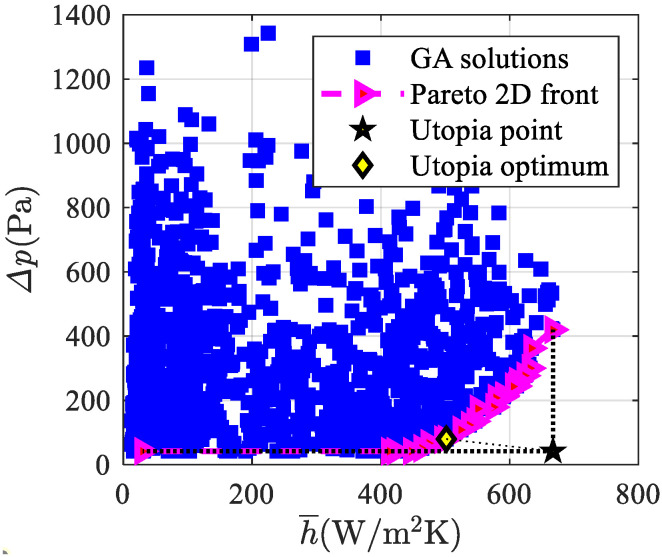
Investigated solutions, Pareto front, and Utopia optimum for **F**_1_(**x**).

The multi-objective optimization for **F**_1_(**x**), **F**_2_(**x**), and **F**_3_(**x**) objective functions is presented in this sub-section. Figure 5 shows the investigated solutions, the Pareto front, and the utopia optimum for **F**_1_(**x**) (see Equation (12)), i.e., the optimum values of the average heat transfer coefficient and pressure drop. The Pareto front spans in the 413 W/m^2^ K–667 W/m^2^ K range of the average heat transfer coefficient and in the 42 Pa–420 Pa range of the pressure drop. The utopia optimum values are $\bar{h}$ = 502 W/m^2^ K and Δ*p* = 80 Pa.

**Figure 6:**
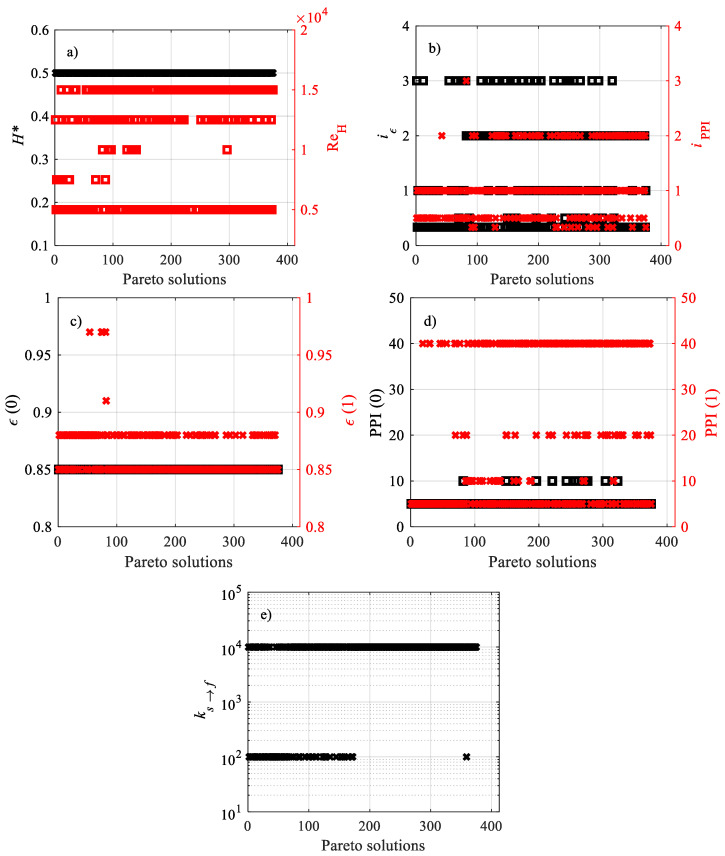
Design variables of the Pareto non-dominated solutions for **F**_1_(**x**): (**a**) H* and Re_H_, (**b**) *i_ε_* and *i*_PPI_, (**c**) *ε*(0) and *ε*(1), (**d**) PPI(0) and PPI(1), (**e**) *k_s__→__f_*.

The design variables of the Pareto non-dominated solutions for **F**_1_(**x**) are reported in Figure 6. Figure 6a,c shows that all Pareto solutions belong to H* = 0.50 and *ε*(0) = 0.85. This occurs because H* = 0.50 characterizes the shortest investigated channel, and it is well known that the local heat transfer coefficient decays along the length. On the other hand, the pressure drop is the smallest, since the length of the channel is the shortest. As for *ε*(0), the largest fraction of highly conductive solid phase close to the wall implies the maximum heat transfer. In contrast, preferential values are presented by variables *ε*(1) in Figure 6c and PPI(0) in Figure 6d, whose optimal values are among the lowest in the investigated domain. The reason why this occurs depends on the PPIs, which are lower in the region far from the wall where heat flux is applied, and which still promote local convection and increase pore velocity. However, the above effect is less marked for *ε*(0), and this means that points at *ε*(1) = 0.88 belong to the Pareto front. As for PPI(0), in Figure 6d, the low permeability and inertial factor (see Table 1) reduce the pressure drop, making lower PPIs optimal for pressure drop-related objective functions. At the same time, some points with 5 PPI are on the Pareto front because interfacial convection is quite weak, too, even if it does not have the same impact of lowering the pressure drop. The spread of the remaining design variables confirms the contrast between the characteristics in **F**_1_(**x**) and the usefulness of multi-objective optimization. When a fixed design variable is so spread, it means that the performance indicators of each objective function are in total contrast with each other. For instance, Re_H_ presents the spread value in Figure 6b, which means that the increasing Reynolds number promotes heat transfer, but has an impact on the pressure drop, too. Having some spread design variables makes the multi-objective optimization very meaningful, since there is no preferential design variable value with which to obtain the set of optimal solutions, i.e., the Pareto front.

**Figure 7:**
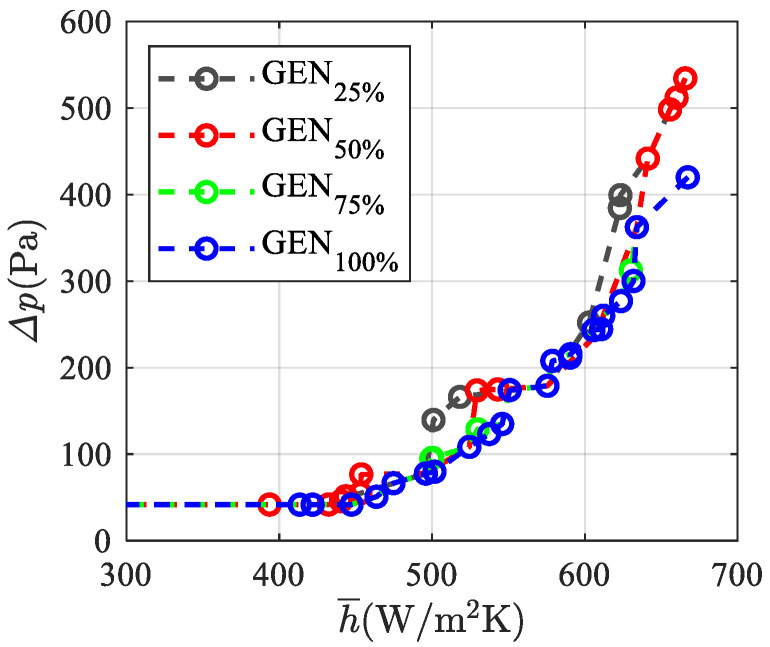
Convergence of the Pareto front and percentages of generations number for **F**_1_(**x**).

The convergence of the Pareto front and various percentages of the generations number (GEN) for **F**_1_(**x**) are presented in Figure 7, which allows us to appreciate how the set of optimum solutions is achieved for different percentages of the generations number. The figure points out that convergence is attained for GEN_75%_.

**Figure 8:**
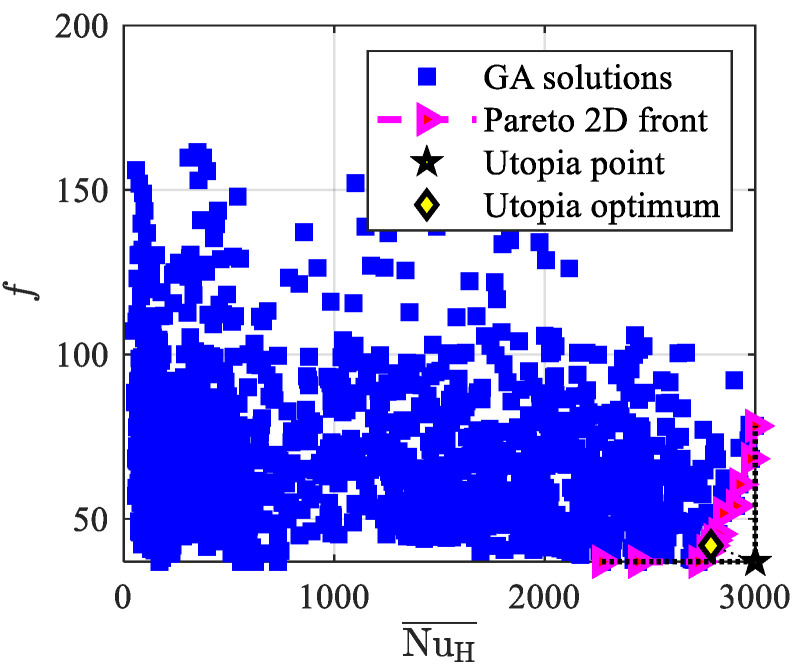
Investigated solutions, Pareto front, and utopia optimum for **F**_2_(**x**).

Figure 8 shows the investigated solutions, the Pareto front, and the Utopia optimum for **F**_2_(**x**), i.e., according to Equation (13), the optimum values of the dimensionless objective functions, the average Nusselt number, and the friction factor. The Pareto front varies in the 2200–3000 range of the average Nusselt number and in the 35–80 range of the friction factor; it starts far from the origin for the Nusselt number and includes few solutions, which provide very high values of $\bar{{\mathrm{Nu}}_{H}}$ with quite small values of f. As it was already achieved for mono-objective optimization, one can compare the herein achieved results to the experimental data reported by Kim et al. [54], who investigated experimentally three aluminum foams (named A, B, and C in the following) under different air mass flow rates in an asymmetrically heated channel. In all foams, the maximum $\bar{{\mathrm{Nu}}_{H}}$ and minimum *f* values were attained at the maximum Reynolds number, which was about 2800. Numerical predictions from the present work can be compared to the experimental results obtained by Kim et al. [54] through the utopia optimum in Figure 8, that is, $\bar{{\mathrm{Nu}}_{H}}$ = 2790 and *f* = 42. Since the comparison must be carried out with different Reynolds numbers, the following expression of PEC_ref_, proposed by Webb and Eckert [53], is used:

(18) PECref=(NuH¯/ReHNuH,ref¯/ReH,ref)(f/fref)1/3,

where the subscript ref indicates data from Kim et al. [54]. If reference is made to the best three couples of $\bar{{\mathrm{Nu}}_{H}}$ and *f* for cases A, B, and C in [54], we find PEC_ref_ = 2.34, 1.87, and 1.60, respectively. Values of PEC_ref_ larger than 1 in all cases highlight the importance of employing both graded foams and numerical optimization in designing the best foam parameters.

**Figure 9:**
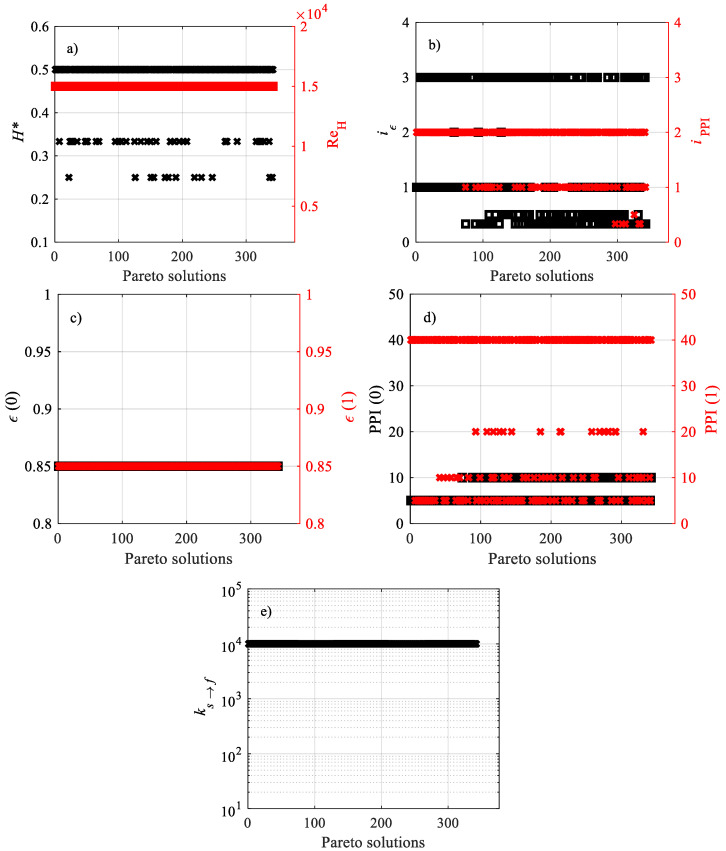
Design variables of the Pareto non-dominated solutions for **F**_2_(**x**): (**a**) H* and Re_H_, (**b**) *i_ε_* and *i*_PPI_, (**c**) *ε*(0) and *ε*(1), (**d**) PPI(0) and PPI(1), and (**e**) *k_s__→__f_*.

The design variables of the Pareto non-dominated solutions for **F**_2_(**x**) are reported in Figure 9. It is worth remarking that, because of the scaling process, the distribution of the above values is somewhat different from that in Figure 6. Figure 9a,e exhibit all the Pareto solutions for the highest Re_H_ = 1.5 10^4^ and *k_s→f_ =* 10^4^, respectively, which means that increasing the Reynolds number increases the heat transfer rate less than the pressure drop. Figure 9c presents all the Pareto solutions for the lowest value of the foam porosity adjacent to the wall, *ε*(0), since the larger fractions of the solid phase are located close to the wall through which the heat enters the foam. The smaller values of PPI(0) in Figure 9d imply a higher impact of the reduction in the pressure drop via the friction factor on the reduction of the heat transfer due to the smallest heat transfer coefficient at y* = 0. It is worth noticing that the smaller the PPI, the lower the volumetric heat transfer. Preferential values are presented by PPI(1) in Figure 9d, whose optimal values are among the highest in the investigated domain.

**Figure 10:**
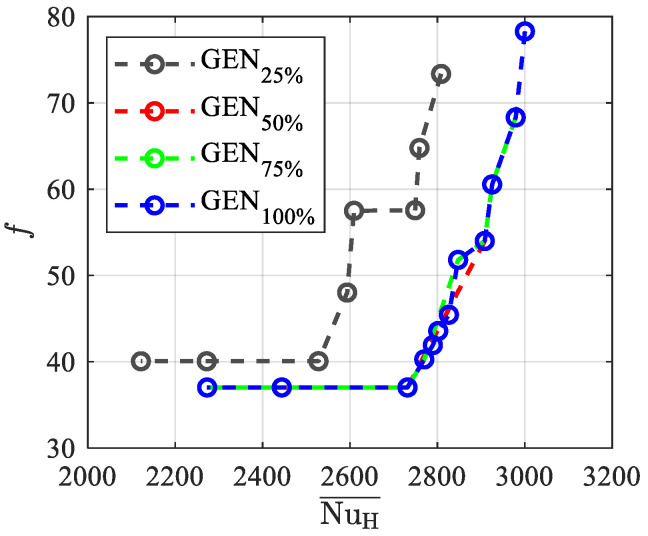
Convergence of the Pareto front and percentages of generations number for **F**_2_(**x**).

The convergence of the Pareto front and various percentages of the generations number (GEN) for **F**_2_(**x**) are presented in Figure 10, which shows a faster convergence than for **F**_1_(**x**), reported in Figure 7; as a matter of fact, one can notice that at about 50% of the generations performed (GE_75%_), the Pareto front approaches the GEN_100_ value.

**Figure 11:**
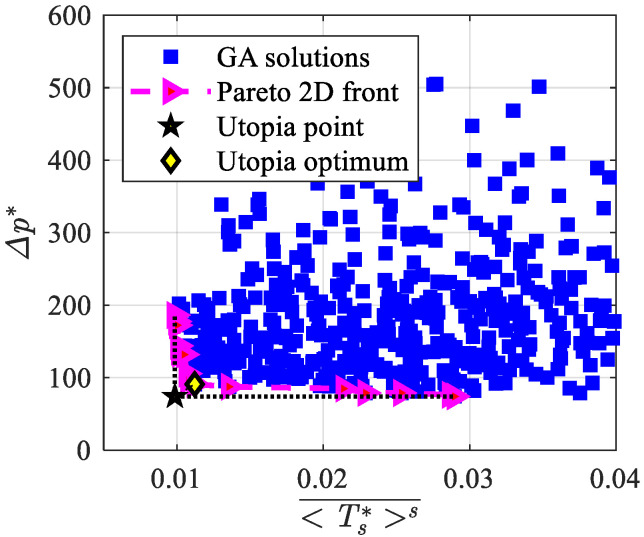
Investigated solutions, Pareto front, and Utopia optimum for **F**_3_(**x**).

Figure 11 shows the investigated solutions, the Pareto front, and the Utopia optimum for **F**_3_(**x**), i.e., according to Equation (14), the optimum values of the dimensionless temperature of the solid phase at y* = 0 and pressure drop objective functions. The Pareto front spans in the 0.010–0.029 range of the dimensionless temperature and in the 74–186 range of the dimensionless pressure drop. The investigated solutions for dimensionless temperatures higher than 0.03 are not reported, since they provide no points on the Pareto front; it means that obtaining smaller surface temperatures implies larger costs.

**Figure 12:**
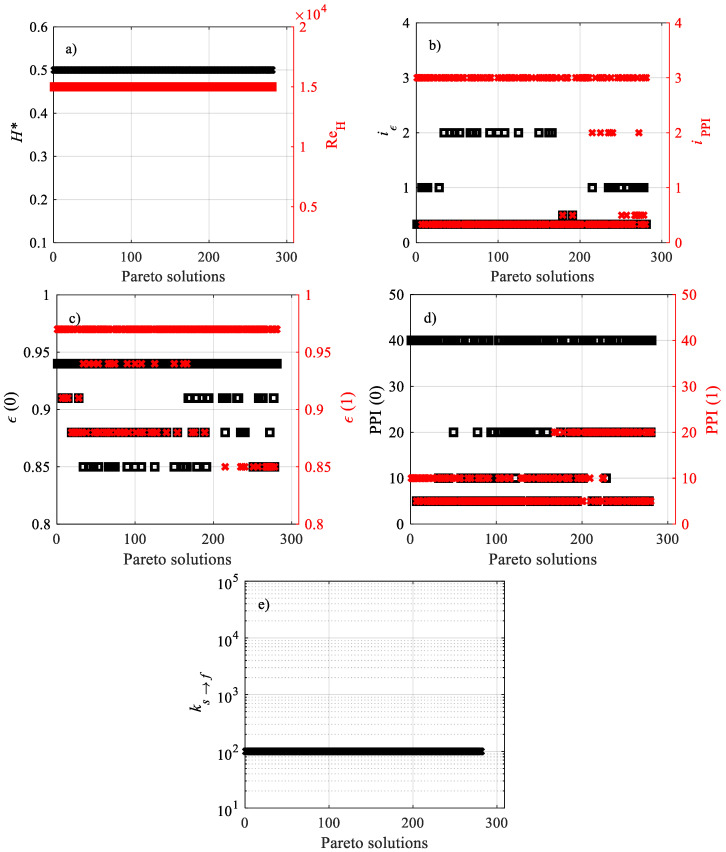
Design variables of the Pareto non-dominated solutions for **F**_3_(**x**): (**a**) H* and Re_H_, (**b**) *i_ε_* and *i*_PPI_, (**c**) *ε*(0) and *ε*(1), (**d**) PPI(0) and PPI(1), (**e**) *k_s__→__f_*.

The design variables of the Pareto non-dominated solutions for **F**_3_(**x**) are reported in Figure 12. Figure 12a shows that all the Pareto solutions belong to H* = 0.5, the same as those for **F**_1_(**x**) and **F**_2_(**x**)**;** the H* maximum value is likely due to the smaller dimensionless temperatures defined in Equation (14). Figure 12a exhibits the highest value of the optimal Reynolds number of all the Pareto solutions, the same as that for **F**_1_(**x**); this is due to the high Reynolds number that enhances heat removal through the solid in the channel. All the Pareto solutions that achieved the minimum *k_s→f_* = 10^2^ value are reported in Figure 12e; as a matter of fact, the lower the thermal conductivity of the solid phase, the lower the dimensionless temperature of the solid phase at the heated wall (see Equation (14)). Looking at Figure 12c, one can remark that at *ε*(1), higher porosities are recommended, since smaller pore velocities (*u_in_*/*ε*) imply smaller pressure drops; on the other hand, at the heated surface, values of *ε*(0) from the Pareto front are lower because lower porosities promote heat removal from the heated wall, though they have undesirable effects on pressure drop. Finally, Figure 12d exhibits PPIs at the heated wall, PPI(0), far higher than those at the adiabatic top wall, PPI(1), since high interfacial convection is required at the heated wall in order to remove heat from it (see Table 1), whereas less heat convection is required at the unheated wall and more emphasis is assumed by the pressure drop, which decreases with increasing PPI, according to the correlations presented in Table 1.

After analyzing the different objective functions **F_j_**(**x**), the design variables shown in Figure 6, Figure 9, and Figure 12, that allow us to optimize them can now be compared. One can remark that, in most cases, the maximum height of the foam, H* = 0.5, optimizes the objective functions because shorter channels generally enhance heat transfer. Unlike Figure 6a, because of the scaling process, the optimizing 1.5 · 10^4^ Reynolds number in Figure 9a and Figure 12a is the same. As for the foam morphology, one can remark that some variability in the power-law indices makes the optimization analysis meaningful. The comparison of **F**_3_(**x**) (Figure 6c–e) with **F**_1_(**x**) (Figure 9c–e) and **F**_2_(**x**) (Figure 12c–e) points out differences in porosity and PPIs for y* = 0 and y* = 1, as well as in thermal conductivity ratios; on the contrary, **F**_1_(**x**) and **F**_2_(**x**) show similar objective functions.

**Figure 13:**
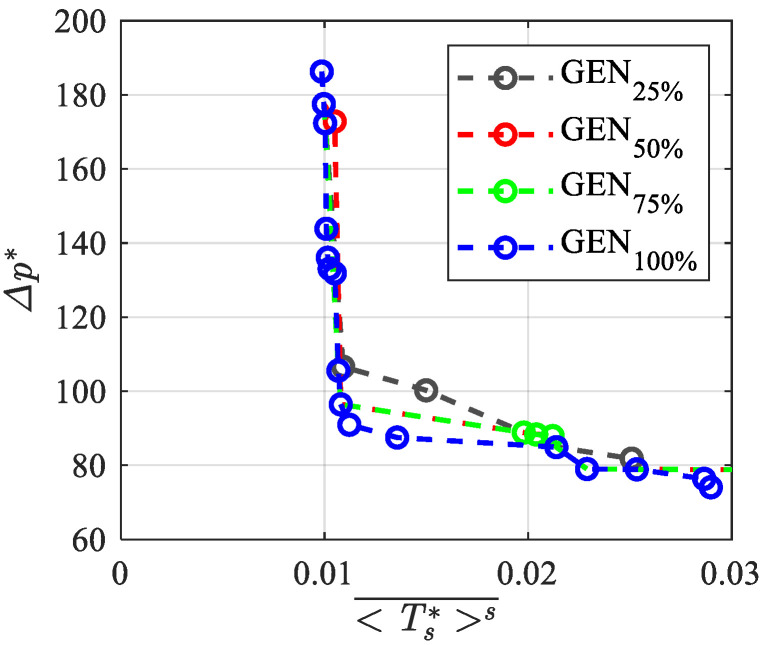
Convergence of the Pareto front and percentages of generation numbers for **F**_3_(**x**).

The convergence of the Pareto front for **F**_3_(**x**) and the various percentages of the generation numbers are presented in Figure 13. The Pareto front slowly approaches the solution GEN_100%_, similarly to what occurs for **F**_1_(**x**) (see Figure 7), whereas the convergence is faster for **F***_2_*(**x**) (see Figure 10), which requires less computational burden. Notably, points with dimensionless temperatures higher than 0.03 for GEN_100%_ are excluded by the optimization algorithm, which finds better solutions during its evolution.

The utopia optimum for multi-objective optimization functions **F_j_**(**x**), the optimum for mono-objective optimization, the optimum from [20], as well as the design variables that allow us to obtain them, are presented in Table 2. The comparison among the different solutions in Table 2 shows that, in all cases, PPI(0) = 5, while *ε*(0) = 0.85 in all cases but 0.91 for **F***_3_*(**x**). This means that foams with low PPIs and low porosities close to the heat source exhibit the best performance. This is justified from a physical point of view, since low PPIs reduce pressure drop and low porosities increase both conduction heat transfer and interfacial heat transfer, since they increase both local velocity and local heat transfer area (see equations in Table 1). On the other hand, it is worth pointing out both that the optimal value of the investigated PPI functions index, *i*_PPI_, is either equal to or lower than 1, and that employing different power-law functions for porosity and PPIs is the best choice.

Finally, all the optimum design variables presented in Table 2 are employed as inputs for the mono- and multi-objective investigated functions. The obtained results for all the investigated objective functions, together with results obtained for the optimum reported in [20], are reported in Table 3. The performance indicators of the optimal solutions reported in Table 3 highlight the importance of choosing the suitable objective function, since, when using the same design variable vector for different objective functions, different optimal performance indicators are obtained. This means that the utopia optimal solution depends also on the chosen objective function, to which attention needs to be paid for its appropriate choice.

## 4. Conclusions

Numerical heat transfer and fluid flow optimization of a channel filled with a graded foam is presented. A rectangular, longitudinal section channel is assumed to be heated uniformly at its bottom wall. Porosity and Pores Per Inch (PPI) morphological parameters are assumed to vary through the domain according to power laws in the heat flow direction. The mathematical model used to predict heat transfer and fluid flow is built up with local thermal non-equilibrium porous media equations.

The velocity of the fluid, the morphology of the foam, and the channel geometry are assumed as design variables. Different optimization approaches are employed:

- A mono-objective approach for the maximization of the Performance Evaluation Criterion (PEC) compared to a uniform foam with the same averaged porosity and PPI;
- Three Pareto two-objective approaches addressing heat transfer coefficient vs. pressure drop (**F**_1_), Nusselt number vs. friction factor (**F**_2_), and dimensionless temperature of the solid phase at the heated wall vs. dimensionless pressure drop (**F**_3_).

The thermal design of the heat sink is optimized using a genetic algorithm (NSGA-II) via the coupling between MATLAB^®^ and COMSOL Multiphysics^®^. The main performance indicators for the optimal solutions are found using the utopia point criterion for multi-criteria decision making, as concerns the multi-objective approaches.

The main conclusions are summarized as follows, and could be considered to be guidelines for the design of such devices based on the mentioned optimal solutions:

- By means of the mono-objective function, a PEC = 1.51 maximum value is achieved, which means a heat transfer efficiency of the optimal simulated graded foams that is 51% higher than that of a uniform foam with the same averaged characteristics. The above reported PEC is higher than the 1.42 achieved in [20], where just a parametric analysis was performed;
- The Pareto front for the multi-objective function **F**_1_(**x**) spans in the 413 W/m^2^ K–667 W/m^2^ K range of the averaged heat transfer coefficient and in the 42 Pa–420 Pa range of the pressure drop. Utopia optimum values are $\bar{h}$ = 502 W/m^2^ K and Δ*p* = 80 Pa;
- For **F**_2_(**x**), the average Nusselt number spans in the 2200–3000 range and the friction factor spans in the 35–80 range. Utopia optimum values are $\bar{{\mathrm{Nu}}_{H}}$= 2790 and *f* = 41.9;
- For **F**_3_(**x**), the dimensionless volume-averaged temperature of the solid phase at the heated wall range is 0.010–0.029 and the dimensionless pressure drop spans between 74 and 186. Utopia optimum values are $\bar{{\langle T_{s}^{*}\rangle }^{s}}$ = 0.011 and Δp* = 91.

The considered performance indicators of the optimal solutions undergo significant variations as a function of the employed optimization approach. The same occurs for the optimal values of the design variables. Therefore, the final user is strongly recommended to carefully choose the objective function depending on actual needs and wills.

Further heat exchanger configurations, such as porous insert channels and so on, should be investigated in the future in order to appreciate the potential of the solutions proposed in the present paper.

## Figures and Tables

**Table 1:** Closing coefficients in the governing equations.

Characteristic	Expression	Source
Permeability, *K* (m^2^); Inertial factor, *C_f_* (-) Strut diameter, *d_s_* (m)	$K\left(y^{*}\right)/{\left[d_{c}\left(y^{*}\right)\right]}^{\;2}=0.00073{\left[1-\varepsilon \left(y^{*}\right)\right]}^{-0.224}{\left[\frac{d_{s}\left(y^{*}\right)}{d_{c}\left(y^{*}\right)}\right]}^{-1.11}$	[46]
	$C_{f}\left(y^{*}\right)=0.00212{\left[1-\varepsilon \left(y^{*}\right)\right]}^{-0.132}{\left[\frac{d_{s}\left(y^{*}\right)}{d_{c}\left(y^{*}\right)}\right]}^{-1.63}$	
	$\frac{d_{s}\left(y^{*}\right)}{d_{c}\left(y^{*}\right)}=\frac{1.18}{G\left(y^{*}\right)}{\left(\frac{1-\varepsilon \left(y^{*}\right)}{3\;\pi }\right)}^{0.5}$	
	$G\left(y^{*}\right)=1-e^{-\frac{1-\varepsilon \left(y^{*}\right)}{0.04}}$	
Cell size, *d_c_* (m)	$d_{c}\left(y^{*}\right)=10^{-3}\left\{-0.921\;\ln\left[\mathrm{PPI}\left(y^{*}\right)\right]+5.3564\right\}$	[47]
Fluid and solid phases effective thermal conductivities, *k_eff,f_* (W/m K); *k_eff,s_* (W/m K)	$k_{eff,f}\left(y^{*}\right)=\varepsilon \left(y^{*}\right)k_{f}$	[48]
	$k_{eff,s}\left(y^{*}\right)=0.329\left[1-\varepsilon \left(y^{*}\right)\right]k_{s}$	
Volumetric heat transfer coefficient, *h_v_* (W/m^3^ K)	$h_{v}\left(y^{*}\right)=\frac{Nu_{v}\left(y^{*}\right)k_{f}}{{\left[d_{c}\left(y^{*}\right)/\varepsilon \left(y^{*}\right)\right]}^{\;2}}$	[49]
	Nuv(y*)=2.864[Rec(y*)] 0.4872[ε(y*)]−5.306	
	$Re_{c}\left(y^{*}\right)=\frac{u_{i}\left[d_{c}\left(y^{*}\right)/\varepsilon \left(y^{*}\right)\right]}{\nu }$	

**Table 2:** Utopia optimum for multi-objective optimization functions **F_j_**(x), optimum for mono-objective optimization, optimum from [20], and design variables.

F_j_(x)	H*	Re_H_	*i_ε_*	*i*_PPI_	*ε*(0)	*ε*(1)	PPI(0)	PPI(1)	*k_s→f_*	Optimum
**F**_1_(**x**) (Equation (12))	0.50	5000	2.00	1.00	0.85	0.85	5	40	10^4^	$\bar{h}$= 502 W/m^2^ K
										Δ*p* = 80 Pa
**F**_2_(**x**) (Equation (13))	0.50	15,000	3.00	1.00	0.85	0.85	5	10	10^4^	$\bar{{\mathrm{Nu}}_{H}}$= 2790
										*f* = 42
**F**_3_(**x**) (Equation (14))	0.50	15,000	0.33	0.33	0.91	0.97	5	5	10^2^	$\bar{{\langle T_{s}^{*}\rangle }^{s}}$= 0.011
										Δp* = 91
PEC (Equation (17))	0.33	15,000	0.33	1.00	0.85	0.97	5	40	10^3^	PEC = 1.51
[20]	0.50	15,000	0.50	0.50	0.85	0.97	5	40	10^4^	PEC = 1.42

**Table 3:** Performance indicators of the optimal solution of the multi-objective optimizations, the mono-objective optimization, and the optimal solution by [20].

F_j_(x)	PEC	$\bar{h}(W/m^{2}\;K)$	*p*(Pa)	$\bar{{\mathrm{Nu}}_{H}}\;$	*f*	$\bar{{\langle T_{s}^{*}\rangle }^{s}}$	Δp*
**F**_1_(**x**) (Equation (12))	1.09	502	80	2243	109	0.686	217
**F**_2_(**x**) (Equation (13))	1.01	624	277	2790	41.9	0.523	84
**F**_3_(**x**) (Equation (14))	1.31	37.9	300	151	45.4	0.011	91
PEC (Equation (17))	1.51	136	706	552	71.3	0.051	214
[20]	1.42	577	530	2361	80.2	0.245	160

## Data Availability

Data of the present research are available upon request to the authors.

## Nomenclature

*C*_*f*_	inertial factor
*c*_*p*_	specific heat capacity (J/kg K)
*d*	diameter (m)
*d*_*s*_	strut diameter (m)
*f*	friction factor
**F_j_**(**x**)	objective function
*G*	geometric function (Table 1)
*h*	heat transfer coefficient (W/m^2^ K)
*h*_*v*_	volumetric heat transfer coefficient (W/m^3^ K)
*H*	foam height (m)
*i*	index (Equations (3) and (4))
*k*	thermal conductivity (W/m K)
*K*	permeability (m^2^)
*L*	foam length (m)
**n**	normal vector
Nu_H_	Nusselt number
Nu_v_	volumetric Nusselt number
*p*	pressure (Pa)
*q*	heat flux (W/m^2^)
Re_c_	cell-size Reynolds number
Re_H_	Reynolds number
*T*	temperature (K)
**u**	velocity vector (m/s)
*u*, *v*	velocity components (m/s)
*W*	foam width (m)
*x*, *y*, *z*	Cartesian coordinates (m)
**Greek letters**	
*ε*	porosity
*ρ*	density (kg/m^3^)
*μ*	dynamic viscosity (kg/m s)
**Subscripts**	
*c*	cell
*eff*	effective
*f*	fluid
*i*	inlet
*ref*	reference
*s*	solid
*unif*	uniform
**Superscripts**	
*	dimensionless
*f*	fluid
*s*	solid
**Other symbols**	
<>	porous media volume average
^*___*^	x and/or y directions average
**Acronyms**	
GA	Genetic Algorithm
PEC	Performance Evaluation Criterion
PPI	Pores Per Inch

## References

[1] Mahjoob S., Vafai K. (2008). A synthesis of fluid and thermal transport models for metal foam heat exchangers. Int. J. Heat Mass Tran..

[2] Avila-Marin A.L. (2011). Volumetric receivers in solar thermal power plants with central receiver system technology: A review. Sol. Energy.

[3] Chen J., Yang D., Jiang J., Ma A., Song D. (2014). Research progress of phase change materials (PCMs) embedded with metal foam (a review). Proc. Mat. Sci..

[4] Lotfizadeh H., Mehrizi A.A., Motlagh M.S., Rezazadeh S. (2015). Thermal performance of an innovative heat sink using metallic foams and aluminum nanoparticles—Experimental study. Int. Comm. Heat Mass Tran..

[5] Ortona A., D’Angelo C., Gianella S., Gaia D. (2012). Cellular ceramics produced by rapid prototyping and replication. Mater. Lett..

[6] Ge C., Priyadarshini L., Cormier D., Pan L., Tuber J. (2018). A preliminary study of cushion properties of a 3D printed thermoplastic polyurethane Kelvin foam. Packag. Technol. Sci..

[7] Xu Z.G., Zhao C.Y. (2015). Experimental study on pool boiling heat transfer in gradient metal foams. Int. J. Heat Mass Tran..

[8] Yang X., Wang W., Yang C., Jin L., Lu T.J. (2016). Solidification of fluid saturated in open-cell metallic foams with graded morphologies. Int. J. Heat Mass Tran..

[9] Udupa G., Rao S.S., Gangadharan K.V. (2014). Functionally graded composite materials: An overview. Proc. Mat. Sci..

[10] Xu F., Zhang X., Zhang H. (2018). A review on functionally graded structures and materials for energy absorption. Eng. Struct..

[11] Stanev L., Kolev M., Drenchev B., Drenchev L. (2014). Open-cell metallic porous materials obtained through space holders-Part I: Production methods. A review. J. Manuf. Sci. E-T ASME.

[12] Yang J., Yang L., Xu C., Du X. (2015). Numerical analysis on thermal behavior of solid–liquid phase change within copper foam with varying porosity. Int. J. Heat Mass Tran..

[13] Chen X., Xia X.L., Meng X.L., Dong X.H. (2015). Thermal performance analysis on a volumetric solar receiver with double-layer ceramic foam. Energy Convers. Manag..

[14] Wang P., Vafai K. (2017). Modeling and analysis of an efficient porous media for a solar porous absorber with a variable pore structure. J. Sol. Energ.-T ASME.

[15] Zaragoza G., Goodall R. (2013). Metal foams with graded pore size for heat transfer applications. Adv. Eng. Mater..

[16] Wang B., Hong Y., Hou X., Xu Z., Wang P., Fang X., Ruan X. (2015). Numerical configuration design and investigation of heat transfer enhancement in pipes filled with gradient porous materials. Energy Convers. Manag..

[17] Xu Z.G., Gong Q. (2018). Numerical investigation on forced convection of tubes partially filled with composite metal foams under local thermal non-equilibrium condition. Int. J. Therm. Sci..

[18] Bai X., Kuwahara F., Mobedi M., Nakayama A. (2018). Forced convective heat transfer in a channel filled with a functionally graded metal foam matrix. J. Heat Trans.-T ASME.

[19] Chen X., Xia X., Sun C., Wang F., Liu R. (2020). Performance evaluation of a double-pipe heat exchanger with uniform and graded metal foams. Heat Mass Transf..

[20] Iasiello M., Bianco N., Chiu W.K.S., Naso V. (2021). The effects of variable porosity and cell size on the thermal performance of functionally-graded foams. Int. J. Therm. Sci..

[21] Safikhani H., Eiamsa-ard S. (2016). Pareto based multi-objective optimization of turbulent heat transfer flow in helically corrugated tubes. Appl. Therm. Eng..

[22] Chamoli S., Yu P., Yu S. (2017). Multi-objective shape optimization of a heat exchanger tube fitted with compound inserts. Appl. Therm. Eng..

[23] Liu C., Bu W., Xu D. (2017). Multi-objective shape optimization of a plate-fin heat exchanger using CFD and multi-objective genetic algorithm. Int. J. Heat Mass Tran..

[24] Du S., He Y.L., Yang W.W., Liu Z.B. (2018). Optimization method for the porous volumetric solar receiver coupling genetic algorithm and heat transfer analysis. Int. J. Heat Mass Tran..

[25] Du S., Ren Q., He Y.L. (2017). Optical and radiative properties analysis and optimization study of the gradually-varied volumetric solar receiver. Appl. Energy.

[26] Zheng Z.J., Li M.J., He Y.L. (2015). Optimization of porous insert configurations for heat transfer enhancement in tubes based on genetic algorithm and CFD. Int. J. Heat Mass Tran..

[27] Siavashi M., Bahrami H.R.T., Aminian E. (2018). Optimization of heat transfer enhancement and pumping power of a heat exchanger tube using nanofluid with gradient and multi-layered porous foams. Appl. Therm. Eng..

[28] Bianco N., Iasiello M., Mauro G.M., Pagano L. (2021). Multi-objective optimization of finned metal foam heat sinks: Tradeoff between heat transfer and pressure drop. Appl. Therm. Eng..

[29] Feng S.S., Kuang J.J., Wen T., Lu T.J., Ichimiya K. (2014). An experimental and numerical study of finned metal foam heat sinks under impinging air jet cooling. Int. J. Heat Mass Tran..

[30] Shi C., Wang M., Yang J., Liu W., Liu Z. (2021). Performance analysis and multi-objective optimization for tubes partially filled with gradient porous media. Appl. Therm. Eng..

[31] Kan K., Chen H., Zheng Y., Zhou D., Binama M., Dai J. (2021). Transient characteristics during power-off process in a shaft extension tubular pump by using a suitable numerical model. Renew. Energy.

[32] Kan K., Yang Z., Lyu P., Zheng Y., Shen L. (2021). Numerical study of turbulent flow past a rotating axial-flow pump based on a level-set immersed boundary method. Renew. Energy.

[33] Whitaker S. (1969). Advances in theory of fluid motion in porous media. Ind. Eng. Chem..

[34] Hill R. (1963). Elastic properties of reinforced solids: Some theoretical principles. J. Mech. Physics Solids.

[35] Vafai K., Tien C.L. (1981). Boundary and inertia effects on flow and heat transfer in porous media. Int. J. Heat Mass Tran..

[36] Vafai K. (1984). Convective flow and heat transfer in variable-porosity media. J. Fluid. Mech..

[37] Amiri A., Vafai K. (1994). Analysis of dispersion effect and non-thermal equilibrium, non-Darcian, variable porosity incompressible flow through porous media. Int. J. Heat Mass Tran..

[38] Kurtbas I., Celik N. (2009). Experimental investigation of forced and mixed convection heat transfer in a foam-filled horizontal rectangular channel. Int. J. Heat Mass Tran..

[39] Hutter C., Büchi D., Zuber V., von Rohr P.R. (2011). Heat transfer in metal foams and designed porous media. Chem. Eng. Sci..

[40] Celik H., Mobedi M., Manca O., Ozkol U. (2017). A pore scale analysis for determination of interfacial convective heat transfer coefficient for thin periodic porous media under mixed convection. Int. J. Num. Meth. Heat Fluid Flow..

[41] Ataei-Dadavi I., Chakkingal M., Kenjeres S., Kleijn C.R., Tummers M.J. (2020). Experiments on mixed convection in a vented differentially side-heated cavity filled with a coarse porous medium. Int. J. Heat Mass Tran..

[42] Seguin D., Montillet A., Comiti J., Huet F. (1998). Experimental characterization of flow regimes in various porous media—II: Transition to turbulent regime. Chem. Eng. Sci..

[43] Della Torre A., Montenegro G., Tabor G.R., Wears M.L. (2014). CFD characterization of flow regimes inside open cell foam substrates. Int. J. Heat Fluid Flow.

[44] Wu Z., Caliot C., Flamant G., Wang Z. (2011). Numerical simulation of convective heat transfer between air flow and ceramic foams to optimise volumetric solar air receiver performances. Int. J. Heat Mass Tran..

[45] Della Torre A., Montenegro G., Onorati A., Tabor G. (2015). CFD characterization of pressure drop and heat transfer inside porous substrates. Energy Procedia.

[46] Calmidi V. (1998). Transport Phenomena in High Porosity Fibrous Metal Foams. Ph.D. Thesis.

[47] Andreozzi A., Bianco N., Iasiello M., Naso V. (2020). Natural convection in a vertical channel with open-cell foams. J. Phys. Conf. Ser..

[48] Iasiello M., Bianco N., Chiu W.K.S., Naso V. (2019). Thermal conduction in open-cell metal foams: Anisotropy and Representative Volume Element. Int. J. Therm. Sci..

[49] Iasiello M., Cunsolo S., Bianco N., Chiu W.K.S., Naso V. (2017). Developing thermal flow in open-cell foams. Int. J. Therm. Sci..

[50] Kornev N., Samarbakhsh S. (2019). Large eddy simulation with direct resolution of subgrid motion using a grid free vortex particle. Int. J. Heat Fluid Flow.

[51] Alcântara Pereira L.A., Oliveira M.A., Moraes P.G., Bimbato A.M. (2020). Numerical experiments of the flow around a bluff body with and without roughness model near a moving wall. J. Braz. Soc. Mech. Sci. Eng..

[52] Dukhan N., Chen K.C. (2007). Heat transfer measurements in metal foam subjected to constant heat flux. Exp. Therm. Fluid Sci..

[53] Webb R.L., Eckert E.R.G. (1972). Application of rough surfaces to heat exchanger design. Int. J. Heat Mass Tran..

[54] Kim S.Y., Kang B.H., Kim J.H. (2001). Forced convection from aluminum foam materials in an asymmetrically heated channel. Int. J. Heat Mass Tran..

